# Defining the Protein Seeds of Neurodegeneration using Real-Time Quaking-Induced Conversion Assays

**DOI:** 10.3390/biom10091233

**Published:** 2020-08-25

**Authors:** Matteo Manca, Allison Kraus

**Affiliations:** Department of Pathology, Case Western Reserve University School of Medicine, Cleveland, OH 44106, USA; mxm1317@case.edu

**Keywords:** RT-QuIC, protein seeds, tau, αSynuclein, prions, neurodegeneration, biomarkers, strains, Alzheimer’s disease, Parkinson’s disease

## Abstract

Neurodegenerative diseases are characterized by the accumulation of disease-related misfolded proteins. It is now widely understood that the characteristic self-amplifying (i.e., seeding) capacity once only attributed to the prions of transmissible spongiform encephalopathy diseases is a feature of other misfolded proteins of neurodegenerative diseases, including tau, Aβ, and αSynuclein (αSyn). Ultrasensitive diagnostic assays, known as real-time quaking-induced conversion (RT-QuIC) assays, exploit these seeding capabilities in order to exponentially amplify protein seeds from various biospecimens. To date, RT-QuIC assays have been developed for the detection of protein seeds related to known prion diseases of mammals, the αSyn aggregates of Parkinson’s disease, dementia with Lewy bodies, and multiple system atrophy, and the tau aggregates of Alzheimer’s disease, chronic traumatic encephalopathy, and other tauopathies including progressive supranuclear palsy. Application of these assays to premortem human biospecimens shows promise for diagnosis of neurodegenerative disease and is an area of active investigation. RT-QuIC assays are also powerful experimental tools that can be used to dissect seeding networks within and between tissues and to evaluate how protein seed distribution and quantity correlate to disease-related outcomes in a host. As well, RT-QuIC application may help characterize molecular pathways influencing protein seed accumulation, transmission, and clearance. In this review we discuss the application of RT-QuIC assays as diagnostic, experimental, and structural tools for detection and discrimination of PrP prions, tau, and αSyn protein seeds.

## 1. Introduction

Neurodegenerative diseases are complex, multifactorial, heterogenous diseases identified by the accumulation of misfolded proteins in the brain. Alzheimer’s disease (AD) is characterized by tau and β-amyloid (Aβ) deposits, Parkinson’s disease (PD) by αSynuclein (αSyn) aggregates, and prion diseases by misfolded prion protein (PrP). Variability in disease progression, clinical presentation, and overlapping disease symptoms complicate diagnosis. Further, neurodegenerative diseases frequently have misfolded protein co-pathologies that have been suggested may contribute to the spectrum of disease heterogeneity. For example, AD can have misfolded αSyn in addition to tau and Aβ aggregates, and Lewy body dementias, such as dementia with Lewy bodies (DLB) and Parkinson’s disease dementia (PDD) can have comorbid tau deposits [1,2]. Targeting the characteristic misfolded proteins as biomarkers provides a biochemical definition of disease to aid definitive diagnosis as well as to help elucidate mechanisms associated with disease processes. This includes understanding how co-occurring misfolded proteins may correlate with the clinicopathological spectrum of disease.

The misfolded proteins of neurodegeneration are capable of faithfully self-amplifying their misfolded structures via seeded polymerization mechanisms. The propagation of structurally matched, highly ordered protein assemblies is initiated by the misfolded “protein seed”. The capacity of misfolded proteins to seed further self-propagation was originally defined in prion diseases and is now a broadly recognized feature of the proteins underlying neurodegenerative diseases including AD and PD. Amplification of protein seeds correlates with progressive disease processes, with protein seeding events occurring before the onset of clinical signs. By definition, “protein seeds” describe the misfolded protein conformers capable of protein-based self-propagation, with the prototypical protein seed being that of prions. PrP prions occur when the largely α-helical conformation of native PrP is converted into a predominant β-sheet structure. Continued, prion-seeded conversion events result in propagation, accumulation, and spread of prions within a host. While prion transmission certainly depends on multiple factors such as prion type, titer, and route of exposure, PrP prions can be naturally and readily transmitted between hosts, as is the case with the readily transmissible prions of chronic wasting disease (CWD) in cervids. However, prions come in a plethora of shapes, sizes, and ability to induce pathological and disease-related events (reviewed in [3]). PrP seeds can represent oligomers [4] or larger fibrillar structures [4,5,6,7], can be either protease sensitive or resistant [8,9], and may or may not be associated with neuropathological outcomes in the brain [3,10,11,12,13,14,15]. The structure(s) of mammalian PrP prions are not yet understood at high resolution and to date, models have been proposed to help explain the biophysical and ultrastructural features observed [16,17,18]. Regardless, while atomic resolution structures of PrP prions remain elusive, distinct structural conformers are thought to dictate prion “strains” [19,20,21], characterized by distinct biochemical profiles, fibrillar ultrastructures, as well as clinical outcomes and associated neuropathology. Tau, Aβ, and αSyn protein seeds have now been described, with evidence that they recapitulate many of the features described for prions. This includes strains and an ability to propagate and spread in experimental models of disease [22,23,24,25,26,27,28,29]. The capacity of protein seeds to self-propagate has been exploited to develop seed amplification technologies. This was first done for PrP prions, but multiple assay platforms now exist to amplify αSyn, tau, and Aβ seeds from biospecimens.

Seed amplification assays include both cell-based and cell-free platforms. Cell-based assays measure the amplification of seeding activity initiated by biospecimens using cells that express tau or αSyn protein fragments fused to fluorescent protein tags. Fluorescence resonance energy transfer (FRET) readouts are used to assess seeded aggregate formation. Cell-based assays are ultrasensitive, able to quantify fM quantities of seeds and typically demonstrate a 3-log dynamic working range [30]. Providing early evidence that tau [30] and αSyn seeds [31] can be detected from human and rodent biospecimens, such seeding assays have further suggested that tau seeding activity occurs prior to clinical AD [32,33].

Both the protein misfolding cyclic amplification (PMCA) and the real-time quaking-induced conversion (RT-QuIC) assays are cell-free, in vitro seed amplification assays. PMCA assays use protein substrates derived from brain homogenates [34] or recombinant protein substrates [35] with cycles of sonication to amplify seeding activity. Readouts typically rely on immunoblot detection of amplified products. PMCA assays are also extremely sensitive, demonstrating the ability to detect pg/mL concentrations of seeds [34,36,37]. In addition, PMCA assays are able to replicate strain-specific characteristics of PrP and αSyn protein seeds [36,38,39]. More recently, the term PMCA has also been used to describe a RT-QuIC like setup in multiwell plates using Thioflavin T (ThT) fluorescence to detect αSyn [40] and Aβ seeds [41]. 

**Figure 1:**
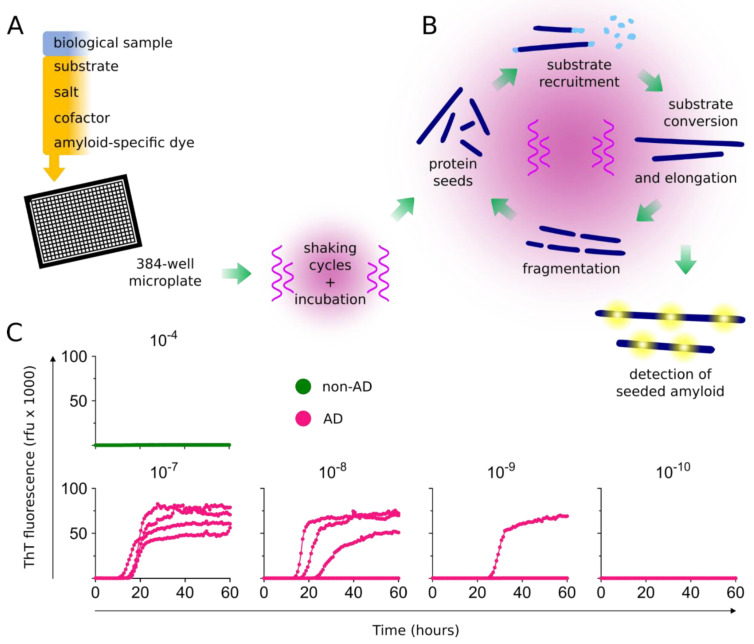
Protein seed amplification using real-time quaking-induced conversion (RT-QuIC) assays. (**A**) Biological samples are added to multiwell plates containing a reaction mixture of substrate(s), salt(s), cofactor(s), and an amyloid-specific fluorescent dye such as Thioflavin T (ThT). Multiwell plates are then incubated, with cycles of shaking and rest and periodic fluorescence readings. (**B**) Protein seeds are exponentially amplified with repeated cycles of substrate recruitment, conversion, and amyloid fibril elongation. Fibrils, generated from cycles of seeded amplification and fibril fragmentation, provide subsequent protein seeds for exponential amplification of the initial seeds. (**C**) Amplification of seeding activity is detected using fluorescent readouts. An example is shown for the amplification of Alzheimer’s disease (AD) tau seeds. Multiwell plates are seeded with the indicated tissue dilution of non-AD (10^−4^) and AD brain tissue homogenates (10^−7^–10^−10^). Each curve represents the ThT amplitude of a single well, run in quadruplicate at each dilution. After 60 h, no seeding activity is observed in the non-AD dilutions, whereas seeding activity is detected in up to 10^−9^ dilutions of AD brain tissue. AD, Alzheimer’s disease; rfu, relative fluorescence units.

RT-QuIC assays use recombinant protein substrates with shaking cycles and incubation to measure seeding activity in a high throughput (96- or 384- multiwell) format. Using the amyloid-specific dye ThT to measure seeded fibril formation, RT-QuICs can detect fg–ag quantities of protein seeds (Figure 1). Biospecimens are used to seed reaction mixtures (Figure 1A) and seeded amyloid formation is monitored over time with fluorescent readouts (Figure 1C). Serial dilutions of the initial sample can be used to determine quantitative seeding doses (SD_50_) with Spearman Kärber analysis [42] as a measure of the specimen dilution at which 50% replicate wells are positive. Optimization of reaction mixtures for substrate(s), buffers, salts, cofactors, detergents, among other components can allow for preferential seed amplification and/or discrimination of seed subtypes [43,44,45,46,47]. Like PMCA, RT-QuIC assays were originally developed for the detection of prions [48,49], and have subsequently been developed for detection of αSyn and tau seeds. Here we discuss the current advances in the development of RT-QuIC assays for tau and αSyn aggregates and how the successes in using RT-QuIC assays for prion diseases might be transferable to other neurodegenerative diseases.

## 2. RT-QuIC Assays as Diagnostic Tools

There is considerable interest in developing biomarkers to classify neurodegenerative disease entities, including those that specifically target the characteristic aggregated proteins. In addition to improving our mechanistic understanding, such markers will certainly be useful to identify and stratify patient cohorts in the development of targeted therapeutics. Disease-specific biomarkers can help identify appropriate patients for clinical trials, as well as to follow the efficacy of therapeutics over time. In addition to specificity, the ideal biomarker is highly sensitive to support early diagnosis, demonstrates a sufficient dynamic range to evaluate clinically relevant quantitative changes with longitudinal studies, and can be measured in easily accessible biofluid or tissue specimens. Prion RT-QuIC assays have been successfully implemented for the diagnosis of prion diseases, with RT-QuIC evaluation of cerebrospinal fluid (CSF) currently being used as part of the diagnostic work-up for sporadic Creutzfeldt-Jakob disease (sCJD) [50,51,52,53,54]. In addition, prion RT-QuIC assays have been adapted for a range of diagnostically relevant human biospecimens including CSF [48,53,55,56], olfactory mucosa [57], eyes [58], and skin [59,60]. The success with prion RT-QuIC assays suggests that further development of the RT-QuIC assays for tau, Aβ, and αSyn seeds may also be applicable for a range of peripheral and biofluid specimens. The comprehensive application of Aβ, tau, and αSyn RT-QuIC assays will likely provide important corollary measures toward understanding protein seeds, potential comorbidities, and differential diagnosis. However, for the purposes of this review, we will focus on the recent developments of tau and αSyn RT-QuIC assays. 

### 2.1. Tau 

Tau is a microtubule-associated protein encoded by the *MAPT* gene. Alternative splicing gives rise to six tau isoforms in the human brain, which are characterized by 0, 1, or 2 N-terminal sequence inserts and either 3 or 4 microtubule binding repeats resulting in classifications as 3-repeat (3R) or 4-repeat (4R) tau, respectively [61,62,63]. Tauopathies are classified by the predominant isoform (3R, 4R, or 3R/4R) that accumulates as aggregates in the brain. Pick’s disease (PiD) is a 3R tauopathy. 4R tauopathies include corticobasal degeneration (CBD), progressive supranuclear palsy (PSP), globular glial tauopathy (GGT), and argyrophilic grain disease (AGD). AD, characterized by both Aβ and tau deposition, and chronic traumatic encephalopathy (CTE) both have tau aggregates comprised of 3R/4R tau isoforms. 

Recent near-atomic cryo electron microscopy (EM) structures have further defined the distinct structural conformers that encode the 3R/4R paired helical and straight tau filaments of AD [64], the 3R/4R filaments of CTE [65], the 3R tau of PiD [66], and the 4R tau of CBD [67,68]. Collectively, these structures indicate 3R, 4R, 3R/4R tau amyloids represent distinct, disease-specific structural conformers and provide evidence for a structural basis of tauopathies [69]. The recent development of tau RT-QuIC assays for the selective detection and distinction of 3R, 4R, and 3R/4R tau aggregates suggest these structural conformers can be specifically and differentially amplified. As such, tau RT-QuIC assays may support tauopathy-specific diagnosis in addition to their ability to discriminate tau-based diseases from other protein misfolding diseases.

The original tau RT-QuIC assay was developed for the 3R tau conformers of PiD [45]. Using a substrate largely based on the microtubule binding repeats of 3R tau, the 3R tau RT-QuIC shows high selectivity for the tau seeds of PiD in brain tissue and postmortem PiD CSF samples. PiD seeds in brain tissue specimens can be detected with billion-fold sensitivity. 

Subsequent tau assay developments led to the first 3R/4R tau RT-QuIC assay for the detection of tau filaments of AD and CTE from brain tissue, known as the AD RT-QuIC [44]. The AD RT-QuIC utilizes a substrate encoding the amino acid sequence incorporated into the amyloid core of AD tau filaments as revealed by cryo-EM [64]. Utilization of the primary sequence of the main protease resistant amyloid core as a basis for substrate design in RT-QuIC has also been used successfully for prion diseases, where the amino acid sequence that comprises the protease resistant amyloid core (residues 90–231) provides a robust substrate for the rapid detection of multiple prion strains including those of sCJD [56]. CTE and AD tau filaments are structurally distinct, with the AD amyloid core arranged as a C-shape [64] and CTE core being characterized by an additional hydrophobic channel enclosing non-proteinaceous cofactors [65]. However, the amino acid sequence incorporated into the amyloid core of both AD and CTE tau filaments is the same. Perhaps not surprisingly, use of a tau substrate comprised of this amino acid sequence supports the amplification of both AD and CTE seeds [44]. Showing ultrasensitive capabilities similar to most RT-QuIC assays, AD RT-QuIC can amplify at least fM quantities of tau seeds, making it also comparable in sensitivity to the cell-based assays for tau seeding activity [30]. AD RT-QuIC is also capable of amplifying the 3R/4R tau aggregates that occur in primary age-related tauopathy (PART), a largely non-clinical pathological description of tau aggregates that accumulate as part of an aging process in the absence of Aβ deposits [70]. How and if the 3R/4R tau seeds of PART may differ from those of AD is still unclear. Regardless, distinguishing non-clinical PART seeds from those of clinical AD, quantitatively or qualitatively, will be important for diagnosis. 

Additional assays have been developed for the detection of 4R tau aggregates [46], as well as a second assay capable of detecting 3R/4R (including AD) but also 3R tau conformers (of PiD), known as the K12 RT-QuIC assay [71]. The 4R RT-QuIC demonstrates preferential selectivity for 4R tauopathies including PSP, CBD, and frontotemporal dementia and parkinsonism linked to chromosome 17 (FTDP-17). 4R RT-QuIC can detect 4R seeds in brain tissue with up to 1000–1,000,000-fold sensitivity [46]. 4R tau seeds are also detectable in PSP and corticobasal syndrome (CBS)/CBD post- and premortem CSF, however with much less sensitivity than that demonstrated for brain [46]. The K12 RT-QuIC demonstrates similar sensitivity to that of the AD RT-QuIC, and is able to detect both 3R seeds, in the case of PiD cases, and 3R/4R tau seeds of AD, CTE, and PART [71]. Both the 4R and K12 RT-QuIC assays are capable of discriminating templating subtypes of disease-specific seeds using readouts of ThT amplitudes and fibrillar assay products. This approach was also successfully used in prion RT-QuIC assays and more recently for discriminating the seeds of PD and multiple system atrophy (MSA) in CSF [36] as discussed further below. 

### 2.2. *α*Synuclein

Synucleinopathies are characterized by the accumulation of αSyn aggregates and include PD, DLB, and MSA. Outside of prion RT-QuIC assays, αSyn RT-QuIC is thus far the most developed RT-QuIC seed amplification platform to aid premortem diagnosis, largely using CSF specimens. 

The application of RT-QuIC for the detection of αSyn seeds was first reported by Alison Green and colleagues. Using CSF, they detected seeding activity from DLB and PD cases with a sensitivity of 92% and 95%, respectively, with 100% specificity [72]. PD cases were clinically confirmed, whereas all other cases examined were clinically and neuropathologically confirmed. Subsequent seed amplification assays, with distinct operational parameters, were reported by the Soto [40] and Caughey groups [73], known as αSyn PMCA and αSyn RT-QuIC, respectively. Using CSF from clinically diagnosed cases, the αSyn PMCA reported a diagnostic sensitivity of 88.5% for PD (*n* = 76), 100% for DLB (*n* = 10), and 80% for MSA (*n* = 10) [40]. Specificity for the unrelated neurologic disease controls (*n* = 65) was 96.9%, whereas that for unrelated and neurodegenerative disease controls was 94% [40]. A comparative study using concurrent analysis with the initial αSyn PMCA and RT-QuIC assays demonstrated 92% concordance with blinded evaluations of CSF from clinically diagnosed PD (*n* = 105) and control cases (*n* = 79) [74]. A comparably more rapid αSyn RT-QuIC was developed by Caughey and colleagues, which demonstrates assay times of 1–2 days [73] versus the 5–13 days required by the Green and Soto assays. The Caughey assay demonstrated a 93% sensitivity and 100% specificity with blinded analysis of 29 synucleinopathy cases (PD and DLB) and 31 controls, including AD [73]. Cases examined in this study were clinically diagnosed, with neuropathological confirmation of select AD and DLB cases where autopsy tissue was available. Collectively, these seed amplification assays indicate that αSyn seeds can be detected with incredible sensitivity and specificity and can be done so reproducibly. This has been further indicated in subsequent studies from multiple groups [75,76].

Important aspects of diagnostic biomarkers include their ability to distinguish clinical syndromes, as well as to be used as potential prognostic indicators. This becomes particularly helpful in the cases where symptoms of disease overlap, such as the discrimination of synucleinopathies from dementias including AD and atypical parkinsonisms such as PSP and CBS. αSyn RT-QuIC was used to evaluate αSyn seeding activity in CSF from uncertain cases of parkinsonism and controls [77]. This study reported a diagnostic accuracy of 84% in distinguishing α-synucleinopathies from non-α-synucleinopathies and controls. While the majority of cases (98%) were not neuropathologically confirmed, the clinical diagnosis was re-evaluated at 3 and 12 years after study inclusion by a repeated structured interview and extensive neurological examination by movement disorder specialists [77]. αSyn seeding activity has also been detected in clinical syndromes that can precede parkinsonism and cognitive impairment including isolated rapid eye movement (REM) sleep behavior disorder (iRBD) and pure autonomic failure (PAF) [78,79]. The initial Green αSyn RT-QuIC study identified αSyn seeding activity in CSF from three REM patients [72]. Another study with a much larger patient cohort examined the seeding activity in CSFs from PD, DLB, MSA, iRBD, and PAF cases [76]. αSyn seeding activity was detected in DLB, PD, but also in iRBD and PAF disorders with an overall sensitivity of 95.3% across the αSyn disorders analyzed [76]. Further evidence suggesting αSyn RT-QuIC can detect αSyn seeds that occur prior to clinical signs of disease comes from a study of genetic carrier cases without disease manifestation. Mutations in leucine-rich repeat kinase 2 (LRRK2) are a common cause of genetic Parkinson’s disease. αSyn seeding activity is detectable in LRRK2-PD patients, as well as a small number of non-manifesting LRRK2 p.G2019S carriers [80]. How early, and with what certainty αSyn RT-QuIC assays can be used for prognosis remains to be ascertained. However, these studies indicate αSyn seeds can provide early biomarkers for uncertain cases and potentially pre-clinical synucleinopathies.

The ideal premortem biomarker is readily measured in easily accessible biospecimens/biofluids. αSyn RT-QuIC demonstrates extraordinary sensitivity for αSyn detection from CSF, however, while CSF can be obtained premortem, such samplings require an invasive lumbar puncture. Thus, for RT-QuIC use in routine preclinical screens, or for longitudinal patient assessment in clinical trials, a more readily accessible peripheral biospecimen may be preferential. αSyn seeding activity can be detected from olfactory mucosa nasal brushings in PD and MSA cases, albeit with incomplete sensitivity (19 of 29 cases) and specificity against PSP and CBD tauopathies [81]. Seeding activity has also been detected in fixed specimens of submandibular salivary glands from DLB and PD autopsy cases [82]. Larger cohorts of pre- and postmortem peripheral biospecimens will be revealing as to how readily αSyn RT-QuIC assays can be applied to peripheral tissues.

## 3. Application of RT-QuIC Assays to Understand Characteristics of Protein Seeds

**Figure 2:**
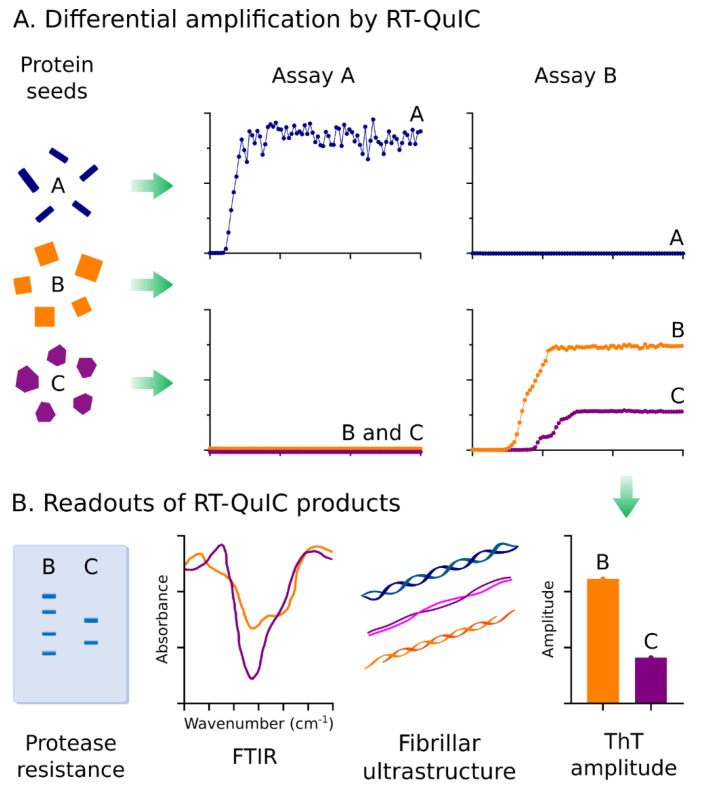
Exploiting properties of protein seeds for differential diagnosis. (**A**) The conformational differences in protein seeds can be exploited for differential amplification using independent RT-QuIC assays that are selective for disease-specific protein seeds. Depicted is a theoretical representation of how two RT-QuIC assays (A&B) can be used to discriminate three different protein seed conformers. Seeds A can be discriminated from B & C using two selective assays capable of amplifying Protein Seed A (Assay A) or B & C (Assay B). Protein seeds B & C can be discriminated by ThT amplitudes within Assay B. (**B**) Conformational-based readouts of RT-QuIC fibrillar byproducts can be used to aid differential diagnosis of protein seeds via RT-QuIC. Fibrillar conformers amplified in RT-QuIC by distinct protein seeds can be reflected via differences in protease resistance, Fourier transform infrared spectroscopy signatures (FTIR), fibrillar ultrastructures (e.g., visualized by electron microscopy), or ThT amplitudes.

The defining characteristics of the seeds detected by αSyn and tau RT-QuIC assays are only beginning to be explored. Seeding activity of 3R/4R tau in AD RT-QuIC is largely sarkosyl insoluble and protease resistant [44]. The selectivity of tau RT-QuIC assays for 3R, 4R, or 3R/4R tau aggregates supports a structural basis for seed propagation. PD and MSA seeds in CSF are able to faithfully replicate as distinct conformers in the αSyn PMCA that are maintained through serial passages of amplification [36]. A growing number of reports indicate that the fluorescent readouts of seed amplification, including lag time and ThT amplitudes, and the seeded fibrillar products together reflect characteristics of the initial seeds in ways that may be helpful both diagnostically and to further define features of disease specific protein seeds. There is much to be learned about the amplification capacities, structural and biophysical properties, and ultimately the pathological outcomes associated with specific protein seeds. While it is unlikely that RT-QuIC assay fibrillar products fully recapitulate all structural features of the initial protein seed (that likely are comprised of longer polypeptide sequences and may include post-translational modifications), sufficient characteristic structural features of the initial seeds are maintained in experimentally measurable ways. Seed conformers can be distinguished by RT-QuIC assay selectivity and seeding kinetics, ThT amplitudes, and structural assessments of the seeded fibrils using techniques such as Fourier transform infrared spectroscopy (FTIR), circular dichroism, electron microscopy, and protease digestion profiles (Figure 2).

### Exploiting Strains for Diagnosis and Dissecting Disease-Related Mechanisms

Prion strains are thought to reflect distinct structural conformers, characterized by differences in fibrillar ultrastructures, protease resistance, and seed amplification properties. Ultimately, different prion strains result in strain-specific neuropathology and clinical disease manifestations. Akin to the strains of prion diseases, distinct strains of αSyn and tau aggregates have been suggested based on apparently different seeding properties, including amplification characteristics and resulting clinical and pathological outcomes [22,23,24,25,26,27,28,29,83]. This is further supported by the diverse, disease-specific amyloid structures recently reported for AD, CTE, PiD, CBD, and MSA amyloids [64,65,66,67,68,84]. An ability to discriminate strains via RT-QuIC or other seed amplification assays is of considerable value toward definitive diagnosis and effective therapeutic development. That is, structural conformers of strains may underlie heterogeneity of disease severity and progression and require different therapeutic agents. In addition, defining strain conformers based on amplification assays allows the opportunity to begin to understand how different αSyn and tau seeds may contribute to disease-related mechanisms.

RT-QuIC assays collectively represent a platform of selective, seed-optimized amplification assays. As such, multiple assays can be used concurrently to qualitatively distinguish distinct seeds. This approach has been used previously to discriminate prion strains of classic and atypical (L-type and C-type) BSE [85,86,87], and classic and atypical (Nor98) strains of scrapie [49,88]. While most prion RT-QuIC assays demonstrate some selectivity for certain prion types, use of a bank vole recombinant substrate allows seeding activity from 28 different types of prions to be detected, suggesting it provides universal detection of prions, regardless of type [88]. This includes use of bank vole to overcome limitations previously observed in the ability to detect reindeer CWD prions [89]. Similarly, use of different tau substrates can confer specificity for different disease-related tau seeds. 3R, 4R, 3R/4R tau seeds are preferentially amplified by the 3R, 4R, or 3R/4R tau RT-QuIC assays, respectively [44,45,46]. αSyn RT-QuIC assays have also demonstrated assay-specific detection capacities of disease-related αSyn aggregates. Detection of the αSyn seeds of MSA, a synucleinopathy characterized by oligodendroglial cytoplasmic αSyn inclusions [90], has varied depending on the αSyn RT-QuIC assay used, with one RT-QuIC demonstrating amplification of MSA seeds [36], and the other not [76]. Interestingly, under assay conditions where MSA seeds are detectable, they can be discriminated from PD seeds evaluated with the same assay by relative ThT amplitudes, and distinct structural features of the seeded fibrils [36]. Regardless, this is reminiscent of the observed seed selectivity with different prion and tau RT-QuIC assays. Incomplete detection of MSA-types within one assay has also been reported [77]. Another study comparing the seeding activity of isolated αSyn from the frontal cortex and substantia nigra pars compacta from either DLB or PD cases showed seeding activity only with αSyn isolated from DLB cases [91]. Thus, under the conditions of αSyn isolation and RT-QuIC assay parameters used, preferential amplification of DLB seeds was achieved. This may suggest further support that DLB and PD αSyn aggregates reflect different conformational strains. However, this study did not observe seeding activity from crude brain homogenates or with all of the DLB-derived αSyn assessed (75% sensitivity in 6/8 cases) which could indicate a more limited sensitivity of this particular RT-QuIC assay [91] compared to other studies that detected αSyn seeding activity from DLB specimens [73,76]. Regardless, collectively these studies suggest that comparison of seeding activities in multiple different RT-QuIC assays can aid strain-specific discrimination of disease-related PrP, tau, and αSyn seeds.

While utilization of multiple RT-QuIC assays concurrently can help discriminate strains, strain subtyping can be achieved within the same RT-QuIC assay. This has been observed as a feature for some prion, αSyn, and tau RT-QuIC assays. A modified second-generation prion RT-QuIC assay using full-length hamster substrate and CSF biospecimens is capable of identifying sCJD subtypes. An evaluation of CSF samples from 2141 patients indicated that different maximum ThT fluorescent amplitudes and lag times for seeded reactions were measured for MM1 versus MM2 and VV1 versus VV2 sCJD cases [50]. Subtyping allowed for the distinction of MM1 from MM2, and VV1 and VV2 with a 95% and 80% probability, respectively. Typing capacities of prion RT-QuIC have also been observed in the bank vole RT-QuIC. Examination of protease-resistant RT-QuIC products indicated the seeded fibrils had distinct disease specific protease sensitivities, further suggesting structural features of the initial seeds are maintained and discernable with RT-QuIC amplification [88].

In the 4R tau RT-QuIC, subtypes of 4R tau seeds from PSP, CBD, and FTDP-17 P301L or N279K mutation cases can be distinguished by differences in maximum ThT amplitudes of seeded reactions, as well as by distinct FTIR signatures of the resulting fibrillar RT-QuIC products [46]. 3R tau seeds of PiD are also distinguishable from 3R/4R tau seeds by comparatively lower ThT amplitudes, and distinct FTIR signatures of seeded products with the K12 tau RT-QuIC [71]. PD and MSA αSyn seeds can similarly be discriminated by differential ThT amplitudes and disease-specific features of the seeded fibrillar products as indicated by differences in fibrillar ultrastructures, FTIR and circular dichroism signatures [36]. One early study by Caughey and colleagues suggested that propagation of PD and DLB seeds resulted in fibrillar products with different ThT amplitudes [73]. However, a second study, albeit using somewhat different RT-QuIC assay parameters, was unable to differentiate PD and DLB seeds by amplitudes [76]. This could reflect that assay-specific conditions are required to maintain subtyping capacities. Regardless, while not all RT-QuIC assays demonstrate subtyping capabilities, seed-specific conformers reflective of strains can be amplified and discriminated in tau, αSyn, and PrP assays.

## 4. Defining Protein Seed Distributions and Networks

RT-QuIC assays are powerful experimental tools with the ability to measure minute quantities of protein seeds from different tissues and biofluids in ways that can be applied to follow seed transmission and accumulation in cell culture and rodent models. Often, assessments of seeding events, seed accumulation, and transmission instead relies on evidence of histologically identifiable misfolded protein accumulation, or immunoblot detection of insoluble or protease resistant material. This might preclude measurements of early seeding events below the detection limits of these experimental methods. With the increasing reports indicating divergent pathogenic and transmission properties of protein seeds (reviewed in [3]), seeding activity measurements can also help detect transmission events of protein seeds that might not otherwise be indicated by clinical disease or pathological outcomes. While histological and immunoblot measures identify accumulation of misfolded proteins, they are not a direct measure of the seeds themselves. Thus, they cannot distinguish seeding events from disruption of proteostatic clearance mechanisms that could also result in the accumulation of misfolded proteins independent of seeding. The application of RT-QuIC and other sensitive seeding assays allows another corollary measurement to dissect mechanisms related to protein seeds and their associated pathological and clinical outcomes in disease models. This will help determine the disease mechanisms directly correlated to seeds, and seed accumulation.

### 4.1. Seeding Activity Assessments of Prion Propagation and Transmission

Prion seeding activity has been used to establish time courses of prion propagation and spread, including very early propagation events following infection [92,93]. RT-QuIC analysis of CSF and brain tissue collected at multiple timepoints from hamsters inoculated with scrapie prions via intracerebral or intratongue routes showed the kinetics with which prion seeding activity can be detected differed depending on the inoculation route [92]. After intracerebral inoculation, seeding activity is detected in the CSF within 1 day, and plateaus within 30 days, whereas brain seeding activity continuously increases up until clinical disease (~75 dpi). Following intratongue inoculation, seeding activity is first detected in the brain and eventually in the CSF albeit only days before onset of clinical signs of disease [92]. A study looking at the kinetics of early PrP propagation following prion microinjection into C57BL/10SnJ mice used RT-QuIC to look at seeding activity at the injection site at 3 and 7 days post-inoculation (dpi). PrP seeds are detectable within 3 days post-inoculation (dpi), and increases in PrP seeds occur by day 7, indicative of new PrP propagation [93]. This suggests that RT-QuIC assays can be applied to track early events in expanding PrP seed networks. In a follow-up study to the initial reports of prion-seeding activity in skin biopsies from sCJD patients [59], RT-QuIC analyses and serial PMCA (sPMCA) was used to determine the kinetics with which prions can be detected in the skin following intracerebral inoculation into rodent models [94]. Skin from thigh, back, and belly was obtained at multiple time points post-infection. sPMCA indicated seeding activity in the skin as early as 2 weeks post inoculation (wpi) in 263K-inoculated hamsters, and 4 wpi in sCJDMM1-inoculated Tg40h mice, which express human PrP. RT-QuIC assay detected prion seeds in the skin at 3 wpi in hamsters and 20 wpi in Tg40h mice [94]. Prion seeding activity was first detected in skin sampled from the ear pinna and the back (3 wpi), and later in thigh skin (9 wpi). Together, these studies provide examples of how RT-QuIC assays can be readily applied to determine kinetics of seed occurrence and spread within tissues.

Measurements of seeding activity have also been used to identify transmission events of prions that do not induce clinical disease or readily detectable pathological changes [3,10,11]. This includes non-pathological prions generated de novo under RT-QuIC conditions, amplified over several rounds, and then inoculated into rodent models to assess the impact of specific amino acid mutations on the transmission and pathogenicity properties of the generated fibrils [10,11]. Non-pathological prions can be capable of logarithmic seed amplification within a host, but are not associated with pathological changes or clinical disease [10,11]. In addition, previous reports indicate that even with pathological prions that initiate clinical disease, prion seeding activity can occur, and sometimes at high levels (10^8^ seeding doses), in brain regions without histologically visible PrP deposits or other neuropathological indications of prion disease such as gliosis or spongiform change [10,95]. Without assessments of seeding activity, the transmission and propagation of non-pathological prions would have been overlooked. Further studies to understand the spectrum of pathogenicity and transmissibility of protein seeds will aid our understanding of neurodegenerative disease-related mechanisms, and RT-QuIC and other seed amplification assays are critical tools to help dissect such pathologically silent seed amplification events.

### 4.2. The Networks of Tau and αSyn Seeds

**Figure 3:**
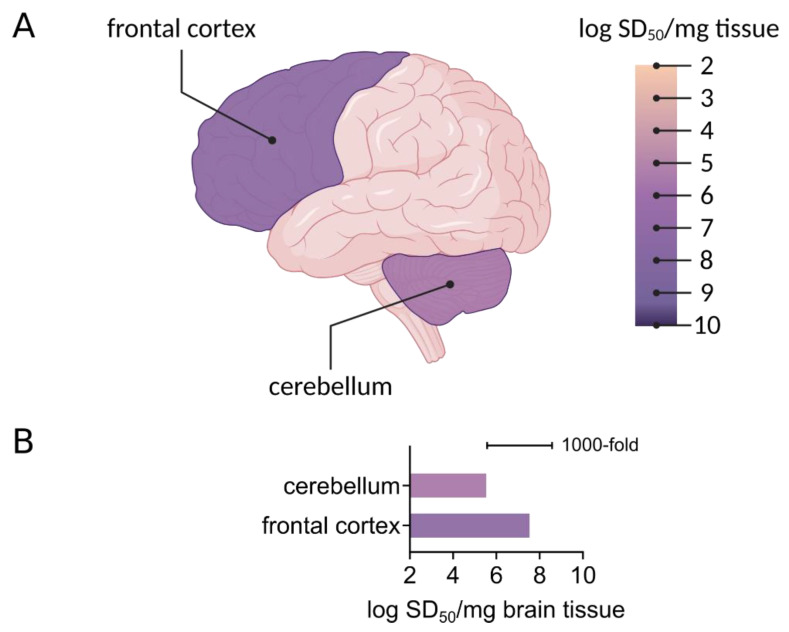
Regional seeding activity detected by RT-QuIC. (**A**) A 3R/4R tau RT-QuIC assay was used to measure seeding activity in the frontal cortex and cerebellum in AD cases. Endpoint dilution analysis was used to determine seeding dose (SD_50_)/mg brain tissue, shown here on a logarithmic scale. Brain regions are colored as per average log SD_50_. Figure created with Biorender.com. (**B**) A bar graph shows the average logarithmic SD_50_ values determined in distinct brain regions as indicated from two AD cases.

The networks and distributions of tau and αSyn protein seeds in preclinical and diseased states are largely not understood. Histological evaluations of tau and αSyn aggregate distribution suggest progressive, staged occurrence of aggregates in ways that correlate with disease [96,97,98]. However, akin to prion seeds, tau seeds are more widespread in brain tissue than suggested by histological evaluations of tau deposits. This includes tau seeding activity in the cerebellum from AD brain tissue ([32,44], Figure 3), which lacks histologically identifiable tau aggregates. Seeding activity is detected in multiple brain regions with (frontal cortex) and without (cerebellum) histologically identifiable tau deposition [44]. Seeding activity in the cerebellum has also been observed with biosensor cell seed amplification assays [32]. Notably, the billion-fold working range of RT-QuIC allows multi-log quantitative distinctions of regional seeding activity, that shows up to 1000-fold differences in seeding activity between the frontal cortex and cerebellum (Figure 3, [44]).

To understand how protein seed networks correlate with neurodegenerative disease processes, when and where seeds occur, and with what other disease markers they are associated with must be defined. As neurodegenerative diseases often include the accumulation of multiple misfolded proteins, this includes how distinct protein seed networks may or may not overlap. From a practical perspective, it becomes important to also understand how comorbid protein seeds might influence seed amplification via RT-QuIC. In the AD RT-QuIC, inclusion of sub- and superstoichiometric quantities of synthetic Aβ oligomers does not influence the kinetics or sensitivity of AD tau seed amplification [44]. However, evidence that mixed seeds might influence RT-QuIC assay readouts is suggested with the observation that inclusion of even small amounts of PiD brain homogenate with AD brain homogenate results in a disproportionate reduction in ThT amplitudes that reflect PiD-like amplification profiles moreso than those of 3R/4R seeds [71]. It is unclear if this effect is mediated by higher seed concentrations in PiD samples, or a preference of the K12 substrate for the PiD-like versus 3R/4R tau conformer. Regardless, further studies to understand if co-occurring protein seeds can influence qualitative or quantitative RT-QuIC assay readouts are warranted.

## 5. RT-QuIC Assays to Determine the Structural Modalities of Seeds and to Test Anti-amyloid Drugs and Disinfectants

Fundamentally, RT-QuIC reactions are real-time kinetic readouts of amyloid fibril formation of the reaction substrate, and the seed-dependent acceleration of such formation. Thus, RT-QuIC assays can also be used to understand molecular features of substrates that influence amyloid formation. For example, RT-QuIC assays have been used to determine amino acids that influence PrP amyloid formation. This includes studies examining the influence of specific proline and lysine residues (within amino acids 101–110) on PrP amyloid formation [99,100]. Disease-associated proline and charge neutralizing substitutions of adjacent lysine residues have a significant impact on the propensity of PrP amyloid formation [99,100]. Both P102 and P105 can be mutated in human genetic prion disease. Replacement of P102, 105, and/or substitution of surrounding lysine residues results in the acceleration of PrP amyloid formation in RT-QuIC [99]. Interestingly, substitution of lysine and proline residues resulted in the formation of PrP amyloids with different transmission and pathogenicity properties [10,11], suggesting RT-QuIC assays can be used to explore molecular features influencing the formation of in vivo seed-competent aggregates.

Use of distinct protein substrates with single amino acid changes can confer selectivity, or stability to an RT-QuIC assay. These characteristics can be exploited both for diagnostic and experimental purposes. One recent study compared the ability of αSyn aggregates from DLB brain tissue and A53T mice to convert either wild-type or A53T mutant αSyn recombinant substrates with RT-QuIC [101]. These findings suggested that αSyn aggregates formed in A53T mice more readily convert A53T recombinant αSyn than wild-type αSyn substrate [101]. It is unclear if the A53T mutant substrate is an absolute requirement for efficient propagation of the A53T αSyn seeds, or if such findings moreso reflect optimized assay condition parameters for A53T seeded amplification.

Prion RT-QuIC analysis has been applied to a wide variety of culture models. RT-QuIC assays have been used to establish prion propagation in organoid models [102], to evaluate the kinetics of prion propagation in slice cultures [103], and to test drug compounds for their ability to influence prion accumulation [103,104]. RT-QuIC was also used to follow the effects of an anti-prion compound in reducing prion seeding activity over time by measuring seeding activity in the urine of prion-infected mice [105]. αSyn RT-QuIC has also been applied to cell culture models. αSyn RT-QuIC has been used to follow uptake and clearance of preformed αSyn fibrils in microglial cell cultures [106]. Sustained seeding activity was measured at multiple time points over 12 h, with no seeding activity being detectable by 24 h [106]. Use of RT-QuIC assays with such systems could help identify signaling pathways, or cellular phenotypes that may influence the cellular kinetics with which particular cell types take up and propagate or degrade protein seeds.

Seeding activity has also been used to establish the efficacy of anti-amyloid activity of disinfectants, including hypochlorous acid (HOCl) [107] and sodium hypochlorite [108] among others. HOCl is a potent disinfectant produced in vivo by immune cells as an innate response to invading pathogens. Synthetic formulations of HOCl have been utilized as disinfectants in laboratory and clinical settings. To test the capacity of HOCl to disinfect prions as well as synthetic αSyn and tau seeds, synthetic formulations of HOCl solutions were pre-incubated with prions, αSyn, or tau fibrils, and seeding activity assessed by RT-QuIC [107]. Preincubation of HOCl with prions, αSyn and tau fibrils significantly reduced their seeding capacity. In the case of prions, this reduction in seeding activity was correlated to conformational changes of the prions and complete abrogation of infectivity as confirmed by bioassay [107]. In a parallel approach, another study used RT-QuIC to establish the minimum incubation times and bleach concentrations required to decontaminate CWD prions in brain homogenates and on stainless steel wires as a surrogate for laboratory, hunting, or meat processing tools that could be contaminated with CWD prions [108]. Thus, use of RT-QuIC assays can complement or in some cases, may even prevent the need for costly and time-consuming bioassays.

### Advantages, Limitations, and Further Development of RT-QuIC Assays

The advantages of RT-QuIC assays have been discussed at length above, and certainly include their ultrasensitivity, high throughput amenability, as well as their ability to discriminate disease specific protein seeds, and recapitulate strain specific conformational features with recombinant protein substrates. Advantages over cell-based seeding assays include an apparently increased dynamic working range, with RT-QuICs having at least a 9-log working range, compared to the 3-log range of cell-based assays, as well as a more accessible laboratory setup that does not require cell culture facilities or a flow cytometer. While these features make RT-QuIC assays well-poised for diagnostic application and use as experimental tools in clinical and laboratory settings, current limitations of RT-QuIC assays must be taken into account.

The ultrasensitive capabilities of RT-QuIC assays allow them to detect protein seeds that occur in even the most minute amounts. Prion RT-QuIC assays can detect ag levels of seeds, which corresponds to 10–40 prion particles [50]. As such, good experimental design is of the utmost importance for diagnostic interpretations, and when applying RT-QuIC assays to experimental cellular and animal models. Like for any highly sensitive technique, aseptic laboratory practices must be employed to mitigate risks of contamination in addition to the routine use of well-vetted controls. Application of RT-QuIC assays to establish kinetics of seed propagation must carefully account for the seed contribution from the initial inoculum to interpret early propagation events. Interpretations of detection sensitivity must be diligently benchmarked against very well-characterized specimens. As RT-QuIC assays have only begun to help define the characteristics of αSyn, tau, and Aβ protein seeds, there is still a limited understanding of the structural conformers that may occur, and therefore the optimal in vitro conditions with which specific seed conformers can propagate. As such, an absence of seeding activity could reflect the absence of seeds, but also a limitation of the specific RT-QuIC conditions to support the amplification of those particular seeds. However, this selectivity for distinct seeds is by no means strictly a limitation, but a feature of RT-QuICs that can be exploited for differential diagnosis as described in this review.

From a diagnostic perspective, it remains to be established what the clinical implications of low levels of seeds are, particularly in specimens from non-symptomatic patients. Careful longitudinal analysis with samples collected prior to, and throughout clinical disease course will be important to help establish the quantitative correlates of seeding activity and clinical disease.

Toward further development of RT-QuIC assays, recent efforts to explore the influence of ionic environment on seed amplification has revealed that the fidelity and sensitivity of seed detection can be drastically altered by inclusion of more weakly or strongly hydrating ions [43]. The Hofmeister ion series, describing an ordered series of anions and cations and their influence on the solubility of proteins [43], was tested over a panel of RT-QuIC assays for PrP, tau, and αSyn seeds. Distinct effects of the series were noted for PrP, tau, and αSyn RT-QuIC assays. 3R/4R tau AD and PrP (hamster and bank vole) RT-QuIC assay fidelity was enhanced in weakly hydrated ions compared to strongly hydrated ones. By contrast, 3R tau, 4R tau, and αSyn RT-QuIC demonstrated inverse and bimodal trends of fidelity, respectively, when correlated to the ordered anion series. Modulation of ionic environment was also successful in improving diagnostic sensitivity. Improvements were noted for multiple assay conditions, and in the case of the 4R RT-QuIC, use of Na_3_Citrate instead of NaCl allowed a million-fold increase in detection sensitivity. Hofmeister effects were further exploited to enhance detection of seeds in plasma, nasal brushings, and ear homogenates. Thus, modulation of ion effects [43], and other RT-QuIC parameters including shaking, incubation temperature, inclusion of beads and cofactors [47] can certainly improve assay fidelity and sensitivity in ways that can be optimized for selective detection of specific seeds. Certainly, empirical determination of the RT-QuIC conditions under which seeds can propagate may aid our understanding of the biochemical factors that influence seeds, and seeding activity.

## 6. Conclusions

Over the last 7 years, efforts have built on premises established for PrP prions to further develop RT-QuIC and related seed amplification assays to amplify Aβ, tau, and αSyn protein aggregates. Just like prion RT-QuIC assays, assays for tau and αSyn have shown extraordinary sensitivity and have further revealed qualitative distinctions in seed detection and faithful propagation of seed characteristics. As the field works to define and understand the clinicopathological heterogeneity of neurodegeneration, the ability of RT-QuIC assays to distinguish protein seeds combined with their ultrasensitive and modulatable detection capabilities underscore the value of RT-QuIC assays as diagnostic and experimental tools.
